# Association between visceral adiposity index and all-cause and cardiovascular mortality in the non-elderly adults

**DOI:** 10.3389/fendo.2025.1523731

**Published:** 2025-02-21

**Authors:** Jiqian Zhang, Ming Li, Tongxin Wang, Wende Tian, Jianqing Ju, Hao Xu

**Affiliations:** ^1^ Graduate School, Beijing University of Chinese Medicine, Beijing, China; ^2^ National Clinical Research Center for Chinese Medicine Cardiology, Xiyuan Hospital, China Academy of Chinese Medical Sciences, Beijing, China; ^3^ Graduate School, China Academy of Chinese Medical Sciences, Beijing, China

**Keywords:** VAI, all-cause mortality, cardiovascular mortality, NHANES, cohort study

## Abstract

**Background:**

The visceral adiposity index (VAI) reflects changes in visceral adipose function and is also linked to cardiometabolic risk. The study aimed to investigate the association between VAI and both all-cause mortality and cardiovascular mortality in the U.S. population aged 20-65 years.

**Methods:**

This study included data from 9,094 American adults aged 20-65 years from the 2009-2018 National Health and Nutrition Examination Survey (NHANES). The exposure variable was VAI, while the outcome variables were all-cause and cardiovascular mortality. The Cox regression model was employed to explore the correlation between VAI and mortality among participants. Restricted cubic splines (RCS) were used to explore the nonlinear associations, and a two-piecewise Cox proportional hazards model was applied on both sides of the inflection point. We used subgroup analyses and interaction tests to further investigate the association between VAI and mortality in different populations. Additionally, time-dependent Receiver Operating Characteristic (ROC) curve analyses were performed to evaluate the capability of VAI in forecasting survival.

**Results:**

During a median follow-up period of 74 months, 251 deaths from all causes and 50 cardiovascular-related deaths were recorded. RCS analyses did not find a nonlinear correlation between VAI and all-cause mortality (P for overall = 0.0006, P for nonlinear = 0.9927) but showed a nonlinear correlation with cardiovascular mortality (P for overall = 0.0010, P for nonlinear = 0.0062). For cardiovascular mortality, when VAI was below the threshold value (2.49), a significant positive association was observed with cardiovascular mortality. When VAI was below 2.49, the risk of cardiovascular mortality increased by 122 percent for each unit increase in VAI (HR=2.22, 95% CI:1.36-3.61). For VAI ≥ 2.49, changes in VAI did not significantly impact cardiovascular mortality risk. In subgroup analyses, the stratified results remained consistent, with no significant interactions observed in any of the subgroups (all P for interaction> 0.05). Furthermore, the areas under the curve (AUC) for 2-, 5-, and 10-year survival rates were 0.82, 0.80, and 0.79 for all-cause mortality and 0.86, 0.86, and 0.82 for cardiovascular mortality, respectively.

**Conclusion:**

VAI was found to have a positive association with all-cause mortality and a nonlinear association with cardiovascular mortality in the non-elderly adults, with a threshold value of 2.49 for cardiovascular mortality.

## 1 Introduction

The global obesity epidemic has emerged as a major public health challenge today, with obesity prevalence doubling across over 70 countries since 1980, while it has continued to rise in most others (1). Obesity can lead to a higher incidence of diabetes, cardiovascular diseases (CVDs), and cancer (2) and is associated with decreased life expectancy (3). CVD remains the leading cause of death worldwide (4), and its burden due to modifiable risk factors such as obesity continues to rise (5). Obesity is responsible for an estimated 4 million deaths worldwide, with over two-thirds of these deaths attributed to CVD (1).

Obesity is defined as an excessive accumulation of body fat, particularly visceral fat (VF) (6). The Framingham Heart Study further highlighted the correlation between visceral adiposity and the risk of developing CVD and cancer (7). In several ethnic groups, a visceral fat area (VFA) of 100 cm² has been established as the optimal cutoff value for detecting individuals at risk for obesity-related disorders (8). Measurements such as Computed Tomography (CT), Magnetic Resonance Imaging (MRI), and Bioelectrical Impedance Analysis (BIA) offer several applications in clinical practice (9). BIA provides accurate and reliable estimates of fat-free mass (FFM) and total body water (TBW) in healthy populations, is faster and relatively inexpensive (10). In addition, the visceral adiposity index (VAI) is also a useful tool for assessing the risk associated with visceral obesity in population-based studies. VAI acts as an indirect measure of visceral adipose function, is an empirical mathematical model calculated using anthropometric indices and functional parameters. It provides a more comprehensive assessment of whole-body fat distribution and has been found to correlate strongly with MRI-measured visceral adiposity (11). The calculation of VAI is simple and fast, which makes it easy to be widely used in routine diagnostics. VAI reflects changes in adipose function (12) and is also linked to cardiometabolic risk (11). A study of common adiposity assessment metrics such as waist circumference, waist-to-hip ratio, and VAI showed that VAI correlated best with adipocytokines and that VAI was able to express altered endocrine function in adipose tissue, including visfatin (13). Of all the indicators, VAI was the only one that showed a negative correlation with adiponectin (13, 14). A study in a population of young women with polycystic ovary syndrome showed that VAI can replace visceral computed tomography as a marker of visceral obesity and predict insulin resistance (15). In patients with non-alcoholic fatty liver disease, VAI was associated with significant fibrosis (16, 17). Furthermore, a higher VAI score is independently associated with steatosis and necroinflammatory activity in genotype 1 chronic hepatitis C patients (18).

VAI has been extensively used to predict the risk of many diseases, including CVD and diabetes (19–22). A J-shaped relationship between VAI and all-cause mortality in the elderly has also been observed (23). However, up to now, few studies have investigated the relationship between VAI and early mortality among individuals aged 20-65 years. This study aimed to explore the association between VAI and all-cause and cardiovascular mortality in individuals aged 20-65 years based on epidemiological evidence. The ultimate goal is to facilitate the early identification of at-risk individuals, promote timely surveillance and intervention for those with visceral obesity, and improve early health outcomes, thereby lowering mortality risk within this demographic.

## 2 Methods

### 2.1 Study population

In this study, participants were selected from the NHANES database, which includes demographic, dietary, examination, and laboratory data to evaluate the health status of the U.S. population. The study received approval from the Research Ethics Review Board of the National Centre for Health Statistics, and all participants provided informed consent (24).

**Figure 1:**
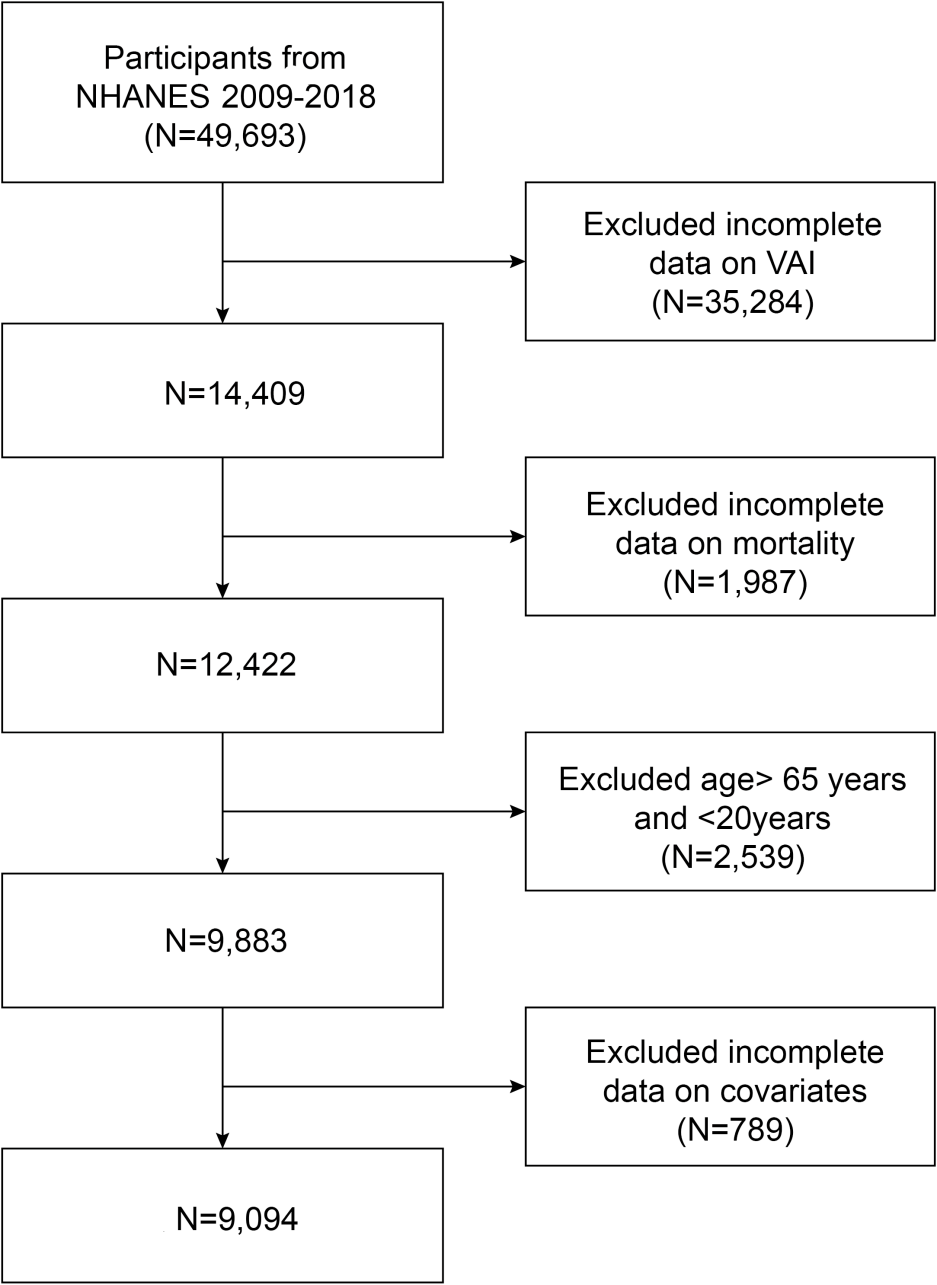
Flow chart of participants selection. NHANES, National Health and Nutrition Examination Survey.

We analyzed data from five consecutive NHANES cycles (2009–2018), encompassing a total of 9,094 participants. We excluded 40,599 participants with incomplete data on VAI, mortality, covariates, and those outside 20 to 65 years old (**Figure 1**).

### 2.2 The measurement of VAI

VAI was calculated using both anthropometric data and biochemical data, including waist circumference (WC), body mass index (BMI), triglyceride (TG), and high-density lipoprotein cholesterol (HDL-C), according to the following formulas:


$$\text{VAI }(\text{male})\;=\text{ WC }(\text{cm})/\;[39.68+1.88*\text{BMI }\left(kg/cm2\right)]\;*\text{TG }(\text{mmol/L})\;/1.03*1.31/\text{HDL-C (mmol/L)}$$

$$\text{VAI }(\text{female})\;=\text{ WC }(\text{cm})/\;[36.58+1.89*\text{BMI }\left(kg/cm2\right)]\;*\text{TG }(\text{mmol/L})\;/0.81*1.52/\text{HDL-C (mmol/L)}$$

### 2.3 Mortality outcomes of the study population

The study used all-cause mortality and cardiovascular mortality as endpoints. Mortality data were obtained from National Death Index (NDI). Follow-up was calculated from the date of the NHANES examination to the date of death or December 31, 2019. Cardiovascular deaths were identified using the International Statistical Classification of Diseases, 10th Revision (ICD-10) codes (I00-I09, I11, I13, and I20-I51) (25).

### 2.4 Covariates

Covariates included age, gender, race/ethnicity, education level, diabetes, hypertension, CVD, low-density lipoprotein cholesterol (LDL-C), and total cholesterol (TC). Participants were classified as having diabetes with one or more of the following criteria: (1) fasting plasma glucose ≥ 7.0 mmol/L or 2-hour oral glucose tolerance test level of ≥ 11.1 mmol/L; (2) HbA1c ≥ 6.5%; (3) self-reported physician-diagnosed diabetes or the use of antidiabetic medications. Participants were defined as having hypertension with one or more of the following criteria: (1) average systolic blood pressure ≥ 140 mmHg; (2) average diastolic blood pressure ≥ 90 mmHg; (3) self-reported physician-diagnosed hypertension or the use of antihypertensive drugs. A history of CVD was defined as self-reported instances of angina pectoris, congestive heart failure, coronary heart disease, heart attack, or stroke.

### 2.5 Statistical analysis

Participants were divided into four groups based on VAI quartiles, the division of upper and lower values was determined according to the actual distribution of the samples (Quartile 1: 0.1002-0.8148; Quartile 2: 0.8149-1.3224; Quartile 3: 1.3230-2.2469; Quartile 4: 2.2475-23.6394). Continuous variables were presented as means ± standard deviation (SD) and compared using the analysis of variance (ANOVA). Categorical variables were presented as frequencies (percentages) and compared using the chi-square test. Cox proportional hazard models were employed to examine the independent association of VAI with all-cause mortality and cardiovascular mortality among participants, followed by further analyses by gender. VAI was analyzed as a continuous variable and in quartile groupings respectively, and results were presented in three models. Model 1 was unadjusted. Model 2 was adjusted for age, gender, and race. Model 3 was adjusted for age, gender, race, education level, diabetes, hypertension, CVD, LDL-C, and TC. Survival probabilities based on baseline VAI levels were evaluated through Kaplan-Meier survival analysis and compared using the log-rank test.

Restricted cubic splines (RCS) were applied to explore the potential nonlinear association between VAI and all-cause mortality and cardiovascular mortality in participants. If the association was nonlinear, a threshold effect analysis was conducted. A two-piecewise Cox proportional hazards model was applied on both sides of the inflection point to examine the association between VAI and both all-cause and cardiovascular mortality. Gender, diabetes, and hypertension variables were considered for stratification and interaction analyses in the subgroup analysis. VAI was used as a continuous variable to explore whether the relationship with mortality was stable across populations. The predictive accuracy of VAI for survival outcomes at various time points was evaluated through time-dependent Receiver Operating Characteristic (ROC) curve analysis. All analyses were performed with R version 4.2 and Empower software. A two-sided P value < 0.05 was considered statistically significant.

## 3 Results

### 3.1 Baseline characteristics of study participants

**Table 1** described the baseline characteristics of enrolled participants with different VAI quartiles in this study. The average age of the subjects was 42.54 years, with 49.02% being male. The mean VAI of the enrolled participants was 1.76 ± 1.45. Compared to the lowest VAI quartile group, participants in the higher VAI quartiles were older, had a larger proportion of non-Hispanic White individuals, and were more likely to have an education level below high school. Additionally, they had higher likelihood of diabetes, hypertension, and CVD. In addition, their LDL-C and TC were usually higher (all P < 0.05).

**Table 1:** Basic characteristics of study participants.

	VAI					
Characteristics	Overall	Quartile 1	Quartile 2	Quartile 3	Quartile 4	*P* value
		(0.1002-0.8148)	(0.8149-1.3224)	(1.3230-2.2469)	(2.2475-23.6394)	
N	9,094	2,274	2,273	2,273	2,274	
Age (years)	42.54 ± 13.21	40.09 ± 13.56	42.02 ± 13.34	43.06 ± 13.05	45.05 ± 12.37	<0.0001
Gender, (%)						0.008
Male	49.02	51.50	47.59	47.03	49.86	
Female	50.98	48.50	52.41	52.97	50.14	
Race/ethnicity, (%)						<0.0001
Non-Hispanic White	63.65	62.03	63.29	62.69	66.61	
Non-Hispanic Black	11.10	16.35	12.76	9.49	5.65	
Mexican American	9.72	6.87	8.78	11.28	12.03	
Other races	15.53	14.75	15.17	16.53	15.70	
Education level, (%)						<0.0001
Less than high school	14.76	10.68	12.48	16.76	19.25	
High school	22.16	18.87	23.84	21.05	24.93	
Greater than high school	63.08	70.45	63.68	62.19	55.83	
Diabetes, (%)						<0.0001
Yes	12.18	4.70	7.89	12.95	23.32	
No	87.82	95.30	92.11	87.05	76.68	
Hypertension, (%)						<0.0001
Yes	31.21	20.52	28.57	33.17	42.83	
No	68.79	79.48	71.43	66.83	57.17	
CVD, (%)						<0.0001
Yes	5.09	2.79	3.76	5.71	8.17	
No	94.91	97.21	96.24	94.29	91.83	
LDL-C (mmol/L)	2.97 ± 0.90	2.62 ± 0.79	2.96 ± 0.81	3.11 ± 0.89	3.21 ± 0.98	<0.0001
TC (mmol/L)	4.95 ± 1.03	4.65 ± 0.93	4.86 ± 0.95	4.99 ± 1.02	5.33 ± 1.09	<0.0001

VAI, visceral adiposity index; CVD, cardiovascular disease; BMI, body mass index; LDL-C, low-density lipoprotein cholesterol; TC, total cholesterol.

### 3.2 Association between VAI and mortality

**Table 2** presented the association between VAI and both all-cause and cardiovascular mortality in the U.S. population aged 20 to 65 years. During a median follow-up period of 74 months, there were a total of 251 all-cause deaths and 50 cardiovascular deaths (Quartile 1: 50 all-cause deaths and 4 cardiovascular deaths; Quartile 2: 59 all-cause deaths and 7 cardiovascular deaths; Quartile 3: 45 all-cause deaths and 14 cardiovascular deaths; Quartile 4: 97 all-cause deaths and 25 cardiovascular deaths). In Model 1, higher VAI was significantly associated with an increased risk of all-cause mortality (HR=1.13, 95% CI:1.06-1.20). This positive correlation between VAI and all-cause mortality remained in Model 2 (HR=1.12, 95% CI:1.05-1.19). Cardiovascular mortality was also positively correlated with VAI. In Models 1 and 2, each unit increase in VAI raised the risk of cardiovascular mortality by 21 percent (HR=1.21, 95% CI:1.08-1.35) and 20 percent (HR=1.20, 95% CI:1.09-1.33). In Model 3, each unit increase in VAI was associated with a 15 percent increase in the risk of cardiovascular death (HR=1.15, 95% CI:1.01-1.30).

**Table 2:** The association between VAI and mortality.

	Model 1		Model 2		Model 3	
	HR (95% CI)	*P* value	HR (95% CI)	*P* value	HR (95% CI)	*P* value
All-cause mortality						
VAI	1.13 (1.06, 1.20)	0.0001	1.12 (1.05, 1.19)	0.0004	1.06 (0.99, 1.14)	0.1192
VAI category						
Quartile1 (0.1002-0.8148)	1 (Ref)		1 (Ref)		1 (Ref)	
Quartile2 (0.8149-1.3224)	1.06 (0.73, 1.54)	0.7695	1.07 (0.73, 1.56)	0.7432	0.96 (0.65, 1.42)	0.8564
Quartile3 (1.3230-2.2469)	0.79 (0.53, 1.19)	0.2599	0.81 (0.54, 1.22)	0.3135	0.71 (0.46, 1.08)	0.1090
Quartile4 (2.2475-23.6394)	1.74 (1.24, 2.45)	0.0015	1.73 (1.22, 2.47)	0.0022	1.32 (0.91, 1.92)	0.1420
P for trend	<0.0001		0.0002		0.0326	
Cardiovascular mortality						
VAI	1.21 (1.08, 1.35)	0.0008	1.20 (1.09, 1.33)	0.0004	1.15 (1.01, 1.30)	0.0394
VAI category						
Quartile1 (0.1002-0.8148)	1 (Ref)		1 (Ref)		1 (Ref)	
Quartile2 (0.8149-1.3224)	1.54 (0.45, 5.25)	0.4930	1.68 (0.49, 5.76)	0.4073	1.48 (0.42, 5.17)	0.5387
Quartile3 (1.3230-2.2469)	3.01 (0.99, 9.15)	0.0519	3.56 (1.16, 10.93)	0.0262	3.06 (0.97, 9.69)	0.0572
Quartile4 (2.2475-23.6394)	5.48 (1.91, 15.75)	0.0016	6.78 (2.31, 19.87)	0.0005	4.45 (1.47, 13.46)	0.0083
P for trend	<0.0001		<0.0001		0.0014	

Model 1: No covariates were adjusted.

Model 2: Age, gender, and race were adjusted.

Model 3: Age, gender, race, education level, diabetes, hypertension, CVD, LDL-C, and TC were adjusted.

HR, Hazard ratio; 95% CI, 95% Confidence Interval; VAI, visceral adiposity index; CVD, cardiovascular disease; LDL-C, low-density lipoprotein cholesterol; TC, total cholesterol.

We further divided VAI into quartiles and similar results were shown. In both Model 1 and Model 2, individuals in the highest VAI quartile showed an increased risk of all-cause mortality (Model 1: HR=1.74, 95% CI:1.24–2.45; Model 2: HR=1.73, 95% CI:1.22–2.47) and cardiovascular mortality (Model 1: HR=5.48, 95% CI:1.91–15.75; Model 2: HR=6.78, 95% CI:2.31–19.87) compared with those in the lowest quartile. In Model 3, compared with participants in the lowest quartile of the baseline VAI, those in the highest quartile had a greater risk of cardiovascular mortality (HR=4.45, 95% CI:1.47–13.46).

We next explored the association between VAI and mortality stratified by gender (**Table 3**). For males, higher VAI was significantly associated with an increased risk of cardiovascular mortality in models 1 and 2 (Model 1: HR=1.18, 95% CI:1.03–1.35; Model 2: HR=1.19, 95% CI:1.04–1.35). The highest VAI quartile showed an increased risk of cardiovascular mortality (Model 1: HR=3.67, 95% CI:1.24–10.93; Model 2: HR=4.96, 95% CI:1.62–15.16; Model 3: HR=3.41, 95% CI:1.06–10.91). For females, the highest VAI quartile showed an increased risk of all-cause mortality (Model 1: HR=4.04, 95% CI:2.03–8.03; Model 2: HR=3.35, 95% CI:1.66–6.75; Model 3: HR=2.20, 95% CI:1.04–4.62). Regarding female cardiovascular mortality, there were no cardiovascular deaths in Quartile 1, VAI was analyzed solely as a continuous variable without quartile grouping. Higher VAI was significantly associated with an increased risk of cardiovascular mortality in models 1 and 2 (Model 1: HR=1.30, 95% CI:1.06–1.59; Model 2: HR=1.32, 95% CI:1.06–1.65).

**Table 3:** Association between VAI and mortality stratified by gender.

	Model 1		Model 2		Model 3	
	HR (95% CI)	*P* value	HR (95% CI)	*P* value	HR (95% CI)	*P* value
Male All-cause mortality						
VAI	1.08 (0.98, 1.18)	0.1142	1.08 (0.99, 1.18)	0.0689	1.00 (0.91, 1.11)	0.9385
VAI category						
Quartile1 (0.1029-0.7917)	1 (Ref)		1 (Ref)		1 (Ref)	
Quartile2 (0.7934-1.3165)	0.87 (0.55, 1.36)	0.5358	0.88 (0.56, 1.39)	0.5943	0.87 (0.54, 1.39)	0.5584
Quartile3 (1.3166-2.2507)	0.73 (0.46, 1.17)	0.1951	0.79 (0.49, 1.27)	0.3221	0.80 (0.48, 1.31)	0.3703
Quartile4 (2.2583-23.6394)	1.24 (0.82, 1.88)	0.3137	1.38 (0.89, 2.12)	0.1466	1.06 (0.67, 1.67)	0.8124
P for trend	0.1317		0.0457		0.5682	
Cardiovascular mortality						
VAI	1.18 (1.03, 1.35)	0.0148	1.19 (1.04, 1.35)	0.0091	1.12 (0.94, 1.33)	0.2132
VAI category						
Quartile1 (0.1029-0.7917)	1 (Ref)		1 (Ref)		1 (Ref)	
Quartile2 (0.7934-1.3165)	1.09 (0.29, 4.04)	0.9025	1.19 (0.32, 4.46)	0.7920	1.15 (0.30, 4.45)	0.8434
Quartile3 (1.3166-2.2507)	2.32 (0.74, 7.30)	0.1495	2.85 (0.90, 9.06)	0.0763	2.94 (0.87, 9.88)	0.0818
Quartile4 (2.2583-23.6394)	3.67 (1.24, 10.93)	0.0193	4.96 (1.62, 15.16)	0.0049	3.41 (1.06, 10.91)	0.0389
P for trend	0.0023		0.0004		0.0155	
Female All-cause mortality						
VAI	1.21 (1.11, 1.33)	<0.0001	1.19 (1.08, 1.31)	0.0007	1.14 (1.02, 1.28)	0.0234
VAI category						
Quartile1 (0.1002-0.8359)	1 (Ref)		1 (Ref)		1 (Ref)	
Quartile2 (0.8360-1.3280)	2.12 (1.01, 4.47)	0.0466	2.03 (0.96, 4.26)	0.0628	1.64 (0.77, 3.49)	0.2011
Quartile3 (1.3292-2.2446)	1.26 (0.56, 2.84)	0.5733	1.13 (0.50, 2.56)	0.7672	0.77 (0.33, 1.79)	0.5416
Quartile4 (2.2456-13.4389)	4.04 (2.03, 8.03)	<0.0001	3.35 (1.66, 6.75)	0.0007	2.20 (1.04, 4.62)	0.0381
P for trend	<0.0001		0.0002		0.0111	
Cardiovascular mortality						
VAI	1.30 (1.06, 1.59)	0.0099	1.32 (1.06, 1.65)	0.0127	1.27 (0.96, 1.68)	0.0896

Model 1: No covariates were adjusted.

Model 2: Age and race were adjusted.

Model 3: Age, race, education level, diabetes, hypertension, CVD, LDL-C, and TC were adjusted.

HR, Hazard ratio; 95% CI, 95% Confidence Interval; VAI, visceral adiposity index; CVD, cardiovascular disease; LDL-C, low-density lipoprotein cholesterol; TC, total cholesterol.

**Figure 2:**
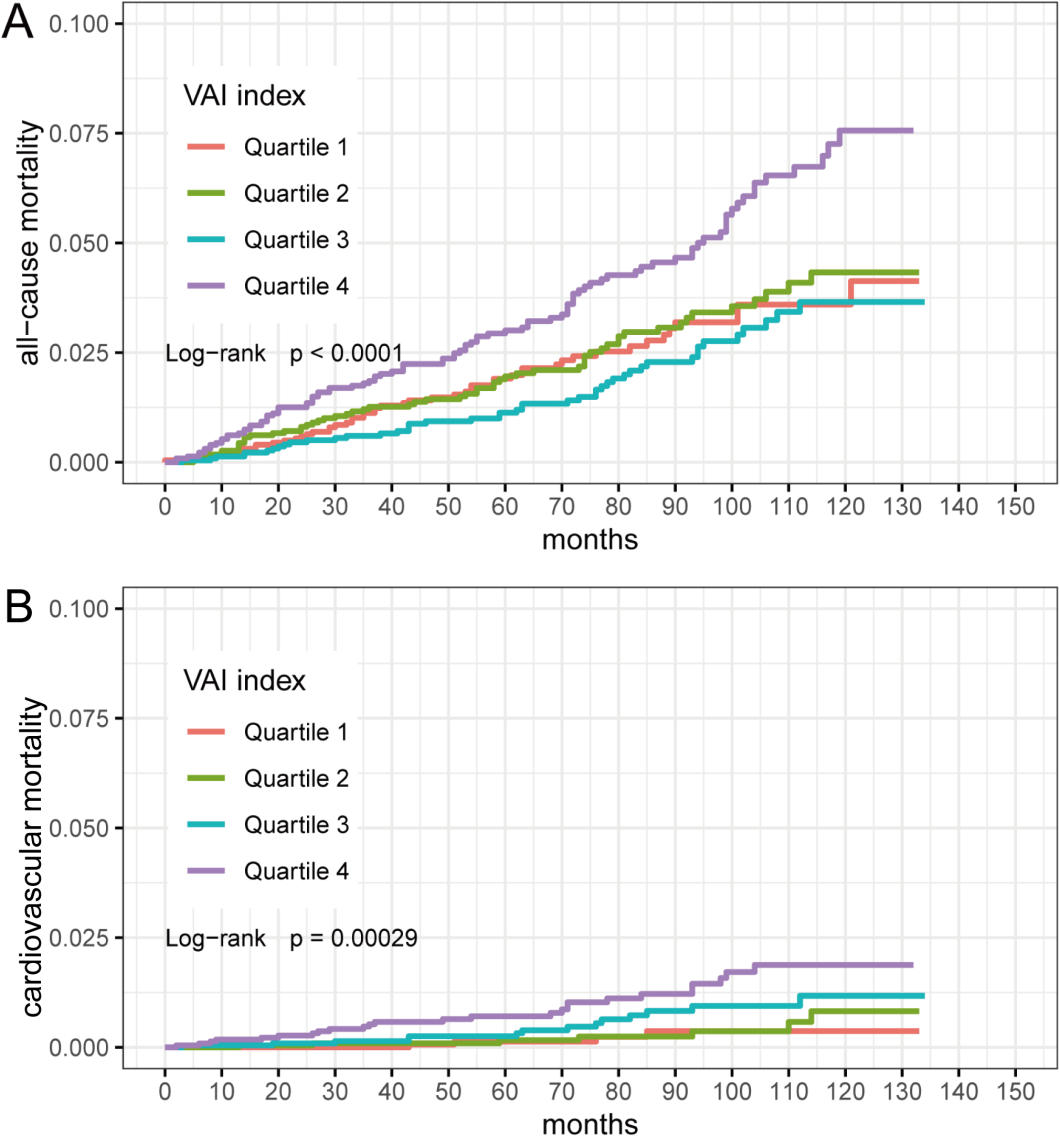
Kaplan-Meier survival curves for VAI quartiles (Quartile 1: 0.1002-0.8148; Quartile 2: 0.8149-1.3224; Quartile 3: 1.3230-2.2469; Quartile 4: 2.2475-23.6394). **(A)** VAI and all-cause mortality; **(B)** VAI and cardiovascular mortality.

We categorized individuals by VAI quartiles (Quartile 1: 0.1002-0.8148; Quartile 2: 0.8149-1.3224; Quartile 3: 1.3230-2.2469; Quartile 4: 2.2475-23.6394). Kaplan-Meier survival curves indicated that different VAI levels were associated with all-cause mortality (log-rank P < 0.0001) and cardiovascular mortality (log-rank P =0.00029) in participants (**Figure 2**). We then performed between-group comparisons for the four groups, and in the relationship between VAI and all-cause mortality, there were significant differences between Quartile 1 compared with Quartile 4 (log-rank P=0.0035), Quartile 2 compared with Quartile 4 (log-rank P=0.0046), and Quartile 3 compared with Quartile 4 (log-rank P=4.9e-05). In the relationship between VAI and cardiovascular mortality, Quartile 1 was significantly different from Quartile 4 (log-rank P=0.0020), and Quartile 2 from Quartile 4 (log-rank P=0.0045).

### 3.3 RCS analysis

**Figure 3:**
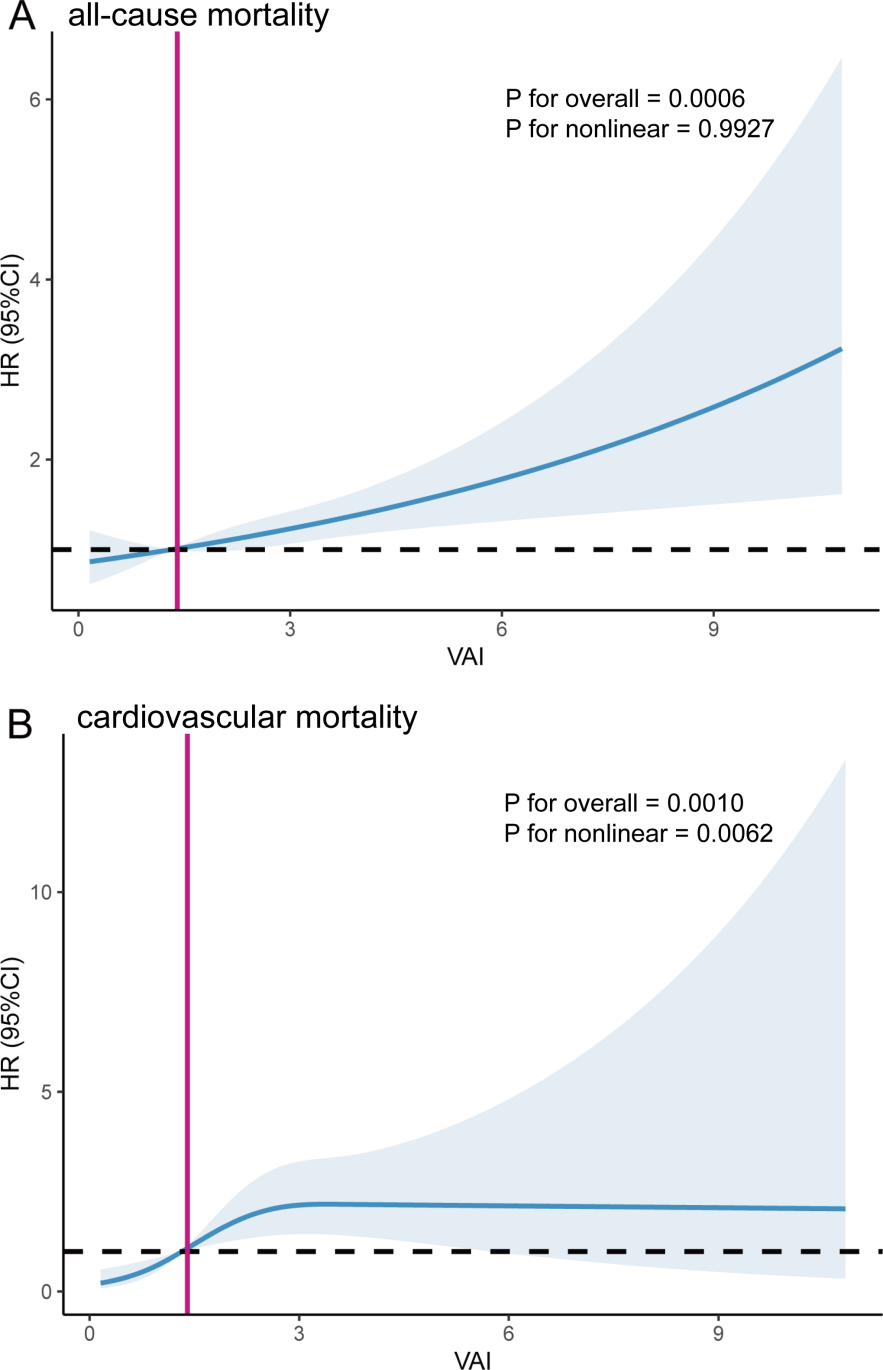
The Restricted cubic spline (RCS) analysis between VAI and all-cause mortality **(A)** and cardiovascular mortality **(B)**. The red line corresponds to the VAI value at HR=1.

We employed RCS analyses to further confirm the potential nonlinear relationship between VAI and mortality from all-cause and cardiovascular. Our study did not identify a nonlinear association between VAI and all-cause mortality (P for overall = 0.0006, P for nonlinear = 0.9927); however, VAI showed a nonlinear association with cardiovascular mortality (P for overall = 0.0010, P for nonlinear = 0.0062) (**Figure 3**).

Participants with a higher VAI had a higher risk of all-cause mortality. Based on the nonlinear association between VAI and cardiovascular mortality, we used a two-piecewise Cox proportional hazard regression model to further fit the association between baseline VAI and mortality (**Table 4**). For cardiovascular mortality, when VAI was below the threshold value (2.49), a significant positive association was observed with cardiovascular mortality. When VAI was below 2.49, the risk of cardiovascular mortality increased by 122 percent for each unit increase in VAI (HR=2.22, 95% CI:1.36-3.61). For VAI ≥ 2.49, changes in VAI did not significantly impact cardiovascular mortality risk. The inflection point for the correlation between VAI and cardiovascular mortality was 1.65 for males and 3.69 for females, showing a similar trend as previously observed.

**Table 4:** Threshold effect analysis of VAI on cardiovascular mortality in participants aged 20-65 years.

	HR (95% CI)	*P* value
Cardiovascular mortality		
Fitting by two-piecewise Cox proportional risk model		
Inflection point	2.49	
VAI < 2.49	2.22 (1.36, 3.61)	0.001
VAI ≥ 2.49	0.89 (0.66, 1.21)	0.465
*P* for log-likelihood ratio	0.004	
**Male**		
Fitting by two-piecewise Cox proportional risk model		
Inflection point	1.65	
VAI < 1.65	4.33 (1.46, 12.81)	0.008
VAI ≥ 1.65	0.87 (0.63, 1.21)	0.418
*P* for log-likelihood ratio	0.008	
**Female**		
Fitting by two-piecewise Cox proportional risk model		
Inflection point	3.69	
VAI < 3.69	2.18 (1.14, 4.16)	0.018
VAI ≥ 3.69	0.78 (0.36, 1.68)	0.523
*P* for log-likelihood ratio	0.062	

HR, Hazard ratio; 95% CI, 95% Confidence Interval; VAI, visceral adiposity index; CVD, cardiovascular disease.

### 3.4 Subgroup analysis

To further investigate the association between VAI and mortality across different populations and evaluate the robustness of the association, we stratified the population according to gender (male/female), diabetes (yes/no), and hypertension (yes/no) (**Table 5**). The stratified results remained consistent, with no significant interactions observed in any of the subgroups (all P for interaction> 0.05).

**Table 5:** Subgroup analysis of the association between VAI and mortality.

Subgroup	HR (95% CI)	*P* value	*P* for interaction
All-cause mortality			
Gender			0.197
Male	1.02 (0.92, 1.13)	0.695	
Female	1.12 (1.01, 1.24)	0.035	
Diabetes			0.479
Yes	1.08 (0.99, 1.19)	0.099	
No	1.03 (0.94, 1.14)	0.528	
Hypertension			0.149
Yes	1.03 (0.94, 1.12)	0.527	
No	1.15 (1.01, 1.31)	0.032	
Cardiovascular mortality			
Gender			0.768
Male	1.13 (0.97, 1.32)	0.110	
Female	1.18 (0.93, 1.50)	0.165	
Diabetes			0.081
Yes	0.97 (0.73, 1.28)	0.808	
No	1.27 (1.07, 1.51)	0.006	
Hypertension			0.289
Yes	1.11 (0.95, 1.30)	0.171	
No	1.33 (1.00, 1.76)	0.047	

VAI was used as a continuous variable to explore whether the relationship with mortality was stable across populations. HR, Hazard ratio; 95% CI, 95% Confidence Interval; VAI, visceral adiposity index; CVD, cardiovascular disease.

### 3.5 The ability of VAI to predict mortality

**Figure 4:**
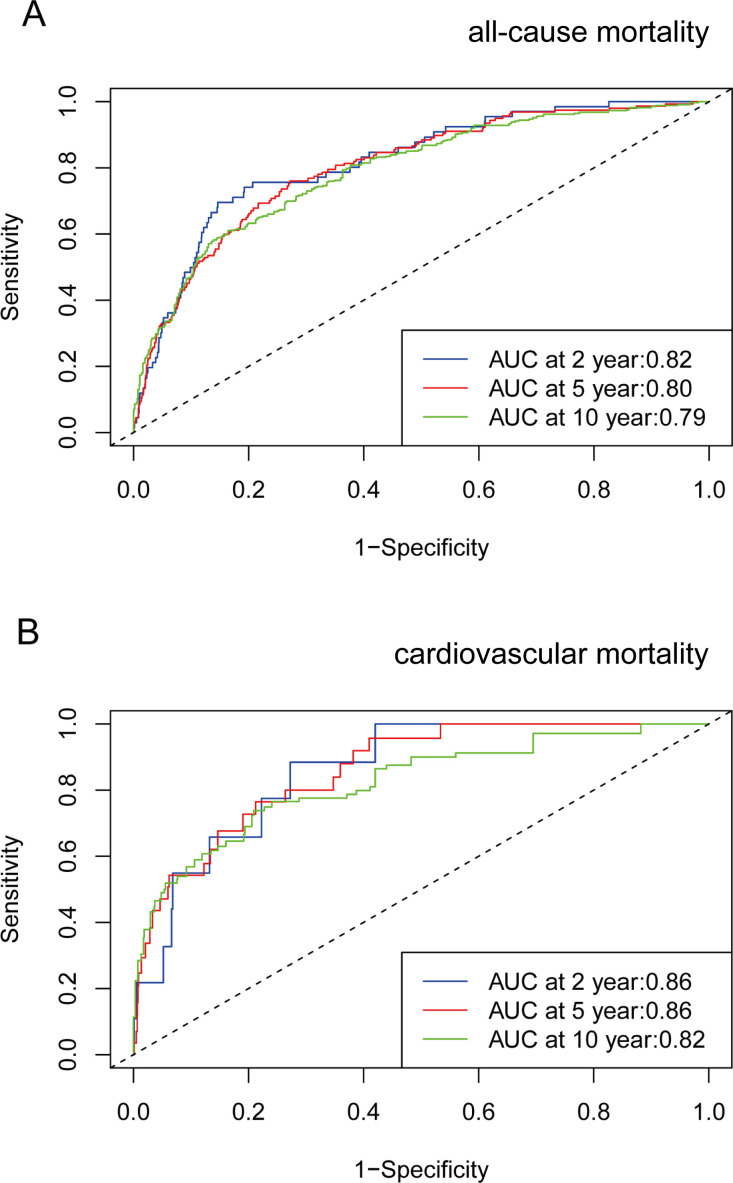
Time-dependent ROC curves and time-dependent AUC values (with 95% confidence band) of VAI for predicting all-cause mortality **(A)** and cardiovascular mortality **(B)**.

Time-dependent ROC curve analysis revealed that the AUC for VAI in predicting all-cause mortality was 0.82 at 2 years, 0.80 at 5 years, and 0.79 at 10 years. Additionally, for cardiovascular mortality, the AUC values were 0.86 at 2 and 5 years, and 0.82 at 10 years (**Figure 4**). These results suggest that VAI maintains consistent predictive accuracy for mortality over various time intervals.

## 4 Discussion

To our knowledge, this study is the first to investigate the association between VAI and both all-cause and cardiovascular mortality in American adults aged 20-65 years. In the prospective cohort study involving 9,094 individuals from the NHANES database, we identified a positive association between VAI and all-cause mortality, as well as a nonlinear association with cardiovascular mortality. A threshold effect analysis identified an inflection point of 2.49 in the correlation between VAI and cardiovascular mortality. In conclusion, our study indicates that VAI is a novel marker for predicting both all-cause and cardiovascular mortality risks in the non-elderly population, potentially contributing to improved prevention and treatment strategies for CVD.

A Canadian study demonstrated that elevated BMI and WC are linked to a greater risk of mortality (26). However, BMI is curvilinear rather than linearly associated with body fat percentage in both men and women (27), and BMI is not a good indicator of fat distribution (28). While WC is considered a valid measure of regional fat distribution, it cannot reliably differentiate between visceral and subcutaneous abdominal fat (12). A study of the NHANES database showed that individuals with a normal BMI yet high body fat (BF) levels displayed a high prevalence of cardiometabolic disorders, metabolic syndrome, and cardiovascular risk factors (29). A community-based study involving participants from diverse racial and ethnic backgrounds demonstrated that visceral obesity is associated with a heightened cardiometabolic risk, regardless of BMI or racial background (30).

This study demonstrates that VAI is a novel biomarker for predicting mortality risk among U.S. individuals aged 20-65. Previous studies have also shown a link between visceral adiposity and all-cause mortality and cardiovascular mortality across different populations. A large UK prospective cohort study demonstrated that higher VAI scores were positively linked to a greater risk of all-cause and cardiovascular mortality (31). A systematic review of 12 cohorts indicated that a large area of abdominal visceral adipose tissue (VAT) is associated with increased all-cause mortality in those aged 65 years or younger (32). Katzmarzyk et al. showed a significant positive correlation between VAT and all-cause mortality in white men and women (33). A study involving 733 Japanese Americans showed that VF was linked to all-cause mortality and obesity-related mortality among Japanese Americans (34). A study based on NHANES III (1988-1994) showed that higher tertiles of VAI are associated with a consistently elevated risk of all-cause and cardiovascular mortality (35). However, our study focused on U.S. individuals between the ages of 20 and 65 years and was designed to explore early mortality in this age group. We utilized data from NHANES 2009–2018, which provided a broader scope and included more recent information.

The relationship between VAI and both all-cause and cardiovascular mortality is influenced by multiple mechanisms. Visceral adipose tissue releases various cytokines, known as adipokines, such as tumor necrosis factor-α (TNF-α), interleukin-6 (IL-6), macrophage migration inhibitory factor, and other protein signals, all of which are strongly linked to inflammatory responses (36, 37). Chronic, low-grade inflammation and macrophage infiltration present in visceral obesity can elevate the risk of CVD (38). Visceral obesity also results in a decreased production of the protective adipokine adiponectin, which has anti-atherosclerotic, anti-diabetic, and anti-inflammatory effects (39). Furthermore, VF accumulation is closely linked to insulin resistance. Adipose tissue contributes to insulin resistance by releasing free fatty acids (FFA), and elevated plasma FFA levels enhance hepatic gluconeogenesis, impairing glucose-stimulated insulin responses (40). Insulin resistance plays a crucial role in the development of metabolic syndrome and CVD (41). Additionally, during VF accumulation, increased FFA levels activate NADPH oxidase, leading to the production of reactive oxygen species (ROS). Increased ROS secretion is also linked to insulin resistance and the progression of atherosclerosis (42). A low VAI can mean that the body’s overall fat reserves are insufficient, which may indicate malnutrition or other health problems, and can also lead to an increased risk of death.

In this study, VAI exhibited a positive association with cardiovascular mortality when its value was below 2.49. However, when the VAI exceeded 2.49, cardiovascular mortality demonstrated a stable and slightly decreasing trend with a consistently high HR and changes in VAI had a nonsignificant effect on cardiovascular disease mortality. Similar to our findings, another study discovered that the dose-response relationship between VAI and angina pectoris, coronary heart disease, and hypertension exhibited a nonlinear parabolic relationship, with the curve beginning to decline when the score reached approximately 3 (43). In the early stages of VAI growth, visceral adipose tissue can increase the risk of cardiovascular disease through a variety of the mechanisms described above, thereby influencing cardiovascular disease mortality. The trend of non-linear association is stable and slightly decreasing in the later period, a phenomenon that may be related to the ‘obesity paradox’. It has been shown that individuals who are overweight or at least mildly obese have a better short- and medium-term prognosis compared to leaner patients with the same CVD (44). Overweight individuals may have greater availability of adipose tissue-associated mesenchymal cells that upon release, could conceivably reduce CVD morbidity. After an acute CVD event, reparative circulating mesenchymal cells (originating from tissues such as adipose tissue, bone marrow, and blood vessels) migrate to the injured myocardial site (45, 46). Adiposity signaling promotes the recruitment of adipocytes from adipose tissue-associate mesenchymal cells (47). It has also been suggested that obese individuals may be protected from atherosclerosis by greater mobilization of endothelial progenitor cells (48). Additionally, in high VAI populations, participants may receive more frequent medical care, pharmacological prophylaxis, and other interventions due to obesity-related conditions, which are often protective factors for potential cardiovascular disease (49). This protective factor applies to patients with diabetes or hypertension as well. In subgroup analyses, we observed an increased risk of cardiovascular mortality in the absence of diabetes or hypertension. The potential reason for this may be that the extent to which lowering hyperglycemia in patients with diabetes reduces atherosclerotic cardiovascular events is unclear (50), whereas patients with diabetes or hypertension are often actively treated with glucose-lowering, anti-hypertensive, lipid-lowering, and even anti-thrombotic therapies, which reduce cardiovascular morbidity and mortality compared to patients without diabetes or hypertension (49).

This study possesses several notable strengths. First, the study population was selected from a nationally representative NHANES cohort, employing a complex multistage probability sampling method. This provided a substantial sample size of 9,094 American adults. Additionally, we further extended this area of research by focusing on the association between VAI and all-cause and cardiovascular mortality in the US population aged 20-65 years. Furthermore, this study further improved the reliability and robustness of the results through stratification and sensitivity analyses. There are also some limitations to this study. First, VAI was developed in a European population and has limitations in the prediction of mortality in other races due to the influence of genetic, epigenetic and environmental factors on fat distribution (51). In individuals with higher levels of visceral fat but lower BMI, the associated risk may be underestimated (52). In future studies, we need to further assess the impact of VAI through broader ethnic and population validation, and the VAI may also complement other obesity assessment metrics for research. Second, menopause can have an impact on women’s cardiometabolic change as estrogen levels decrease (53). Women after menopause were found to have a higher probability of developing CVD (54). Thus, the use of hormone replacement therapy also affects cardiovascular outcome events. In addition, although we have adjusted for multiple covariates, we were unable to completely exclude all potential confounders. Finally, the limitations of the NHANES database made it difficult for us to delve into the dynamic features of the association between VAI and health outcomes. The association of VAI with mortality from other causes also needs to be further explored.

## 5 Conclusion

Our results suggest that VAI serves as a cost-effective and valuable biomarker for assessing the risk of all-cause and cardiovascular mortality in people aged 20-65 years in the US. VAI had a positive association with all-cause mortality and a nonlinear association with cardiovascular mortality. Therefore, we advocate early monitoring and intervention of VAI in the non-elderly population to improve early health and reduce mortality risk in this age group.

## Data Availability

The original contributions presented in the study are included in the article/Supplementary Material. Further inquiries can be directed to the corresponding authors.
